# Supplementary material for: Multi‐dataset identification of innovative feature genes and molecular mechanisms in keratoconus
Source: J Cell Mol Med. 2024 Sep 19;28(18):e70079. doi: 10.1111/jcmm.70079 (PMC11412914; doi:10.1111/jcmm.70079)
Supplement: Supplementary file 2 — Material S2. [file JCMM-28-e70079-s002.zip › 7.GSEA analysis/3.DBNDD1/KEGG_ARGININE_AND_PROLINE_METABOLISM.html]

Details for gene set KEGG\_ARGININE\_AND\_PROLINE\_METABOLISM[GSEA]

|  || Dataset | DBNDD1.group.cls#h\_versus\_l |
| Phenotype | group.cls#h\_versus\_l |
| Upregulated in class | h |
| GeneSet | KEGG\_ARGININE\_AND\_PROLINE\_METABOLISM |
| Enrichment Score (ES) | 0.24174634 |
| Normalized Enrichment Score (NES) | 0.8577626 |
| Nominal p-value | 0.6768916 |
| FDR q-value | 1.0 |
| FWER p-Value | 1.0 |
Table: GSEA Results Summary

  

Fig 1: Enrichment plot: KEGG\_ARGININE\_AND\_PROLINE\_METABOLISM      
 Profile of the Running ES Score & Positions of GeneSet Members on the Rank Ordered List

  

| SYMBOL | TITLE | RANK IN GENE LIST | RANK METRIC SCORE | RUNNING ES | CORE ENRICHMENT || 1 | P4HA3 | na | 571 | 0.434 | 0.0591 | Yes |
| 2 | AGMAT | na | 645 | 0.421 | 0.1305 | Yes |
| 3 | MAOB | na | 696 | 0.415 | 0.2014 | Yes |
| 4 | PRODH2 | na | 1496 | 0.336 | 0.2369 | Yes |
| 5 | CKM | na | 2879 | 0.256 | 0.2417 | Yes |
| 6 | NOS3 | na | 4813 | 0.186 | 0.2184 | No |
| 7 | GAMT | na | 5079 | 0.178 | 0.2417 | No |
| 8 | ARG2 | na | 6137 | 0.147 | 0.2368 | No |
| 9 | OTC | na | 6985 | 0.125 | 0.2341 | No |
| 10 | ASL | na | 7766 | 0.108 | 0.2305 | No |
| 11 | ACY1 | na | 10397 | 0.066 | 0.1661 | No |
| 12 | ALDH4A1 | na | 12098 | 0.046 | 0.1252 | No |
| 13 | GLS2 | na | 12449 | 0.043 | 0.1225 | No |
| 14 | ALDH3A2 | na | 12835 | 0.040 | 0.1183 | No |
| 15 | ASS1 | na | 13633 | 0.033 | 0.1011 | No |
| 16 | GOT2 | na | 13748 | 0.032 | 0.1034 | No |
| 17 | PYCR2 | na | 13937 | 0.030 | 0.1033 | No |
| 18 | GLUD1 | na | 15404 | 0.020 | 0.0645 | No |
| 19 | CKMT1A | na | 16380 | 0.013 | 0.0387 | No |
| 20 | NAGS | na | 16536 | 0.012 | 0.0364 | No |
| 21 | GLUL | na | 17464 | 0.006 | 0.0108 | No |
| 22 | SMS | na | 18350 | 0.000 | -0.0147 | No |
| 23 | ALDH7A1 | na | 18637 | -0.002 | -0.0226 | No |
| 24 | CPS1 | na | 19099 | -0.005 | -0.0350 | No |
| 25 | MAOA | na | 19119 | -0.005 | -0.0346 | No |
| 26 | AMD1 | na | 19469 | -0.008 | -0.0433 | No |
| 27 | LAP3 | na | 19562 | -0.009 | -0.0443 | No |
| 28 | ALDH18A1 | na | 19781 | -0.011 | -0.0487 | No |
| 29 | GOT1 | na | 19996 | -0.013 | -0.0526 | No |
| 30 | SAT2 | na | 20089 | -0.014 | -0.0528 | No |
| 31 | SRM | na | 20771 | -0.020 | -0.0690 | No |
| 32 | GLS | na | 21167 | -0.023 | -0.0763 | No |
| 33 | ALDH1B1 | na | 22410 | -0.036 | -0.1058 | No |
| 34 | CKB | na | 22753 | -0.040 | -0.1086 | No |
| 35 | ALDH9A1 | na | 22786 | -0.041 | -0.1025 | No |
| 36 | GLUD2 | na | 23554 | -0.050 | -0.1158 | No |
| 37 | ARG1 | na | 24337 | -0.061 | -0.1277 | No |
| 38 | GATM | na | 24453 | -0.062 | -0.1202 | No |
| 39 | PRODH | na | 24945 | -0.070 | -0.1221 | No |
| 40 | OAT | na | 25010 | -0.071 | -0.1116 | No |
| 41 | SAT1 | na | 25206 | -0.074 | -0.1044 | No |
| 42 | CKMT2 | na | 25380 | -0.077 | -0.0960 | No |
| 43 | P4HA1 | na | 27650 | -0.122 | -0.1402 | No |
| 44 | AOC1 | na | 28210 | -0.135 | -0.1327 | No |
| 45 | ODC1 | na | 28272 | -0.137 | -0.1105 | No |
| 46 | DAO | na | 28346 | -0.139 | -0.0883 | No |
| 47 | P4HA2 | na | 28667 | -0.148 | -0.0717 | No |
| 48 | PYCR1 | na | 28839 | -0.152 | -0.0501 | No |
| 49 | AZIN2 | na | 31322 | -0.233 | -0.0810 | No |
| 50 | NOS2 | na | 31398 | -0.236 | -0.0420 | No |
| 51 | ALDH2 | na | 33235 | -0.347 | -0.0344 | No |
| 52 | NOS1 | na | 34107 | -0.444 | 0.0179 | No |
Table: GSEA details [plain text format]

  

Fig 2: KEGG\_ARGININE\_AND\_PROLINE\_METABOLISM      
 Blue-Pink O' Gram in the Space of the Analyzed GeneSet

  

Fig 3: KEGG\_ARGININE\_AND\_PROLINE\_METABOLISM: Random ES distribution      
 Gene set null distribution of ES for **KEGG\_ARGININE\_AND\_PROLINE\_METABOLISM**
